# Epidemiology, co-infection, and seasonal patterns of respiratory tract infections in a tertiary care center in Saudi Arabia between 2021 and 2022

**DOI:** 10.3389/fpubh.2025.1492653

**Published:** 2025-04-02

**Authors:** Nabeel Alzahrani, Ahmed Alshehri, Ali Alshehri, Sameera Al Johani

**Affiliations:** ^1^Department of Clinical Laboratory Sciences, College of Applied Medical Sciences, King Saud bin Abdulaziz University for Health Sciences, Riyadh, Saudi Arabia; ^2^King Abdullah International Medical Research Center, Riyadh, Saudi Arabia; ^3^Division of Microbiology, Department of Pathology and Laboratory Medicine, Ministry of the National Guard-Health Affairs, Riyadh, Saudi Arabia

**Keywords:** infectious disease epidemiology, multiplex PCR, respiratory viruses, seasonality, Saudi Arabia

## Abstract

**Objectives:**

To investigate the etiology and epidemiological trends of respiratory tract infections (RTIs) during the COVID-19 pandemic in Saudi Arabia, focusing on age and seasonality.

**Methods:**

We conducted a retrospective analysis of 19,509 respiratory specimens collected from January 2021 to December 2022 at King Abdulaziz Medical City, Riyadh, using the BioFire Filmarray Respiratory Panel 2.1 plus kit.

**Results:**

Of the analyzed specimens, 53.3% (10,406) tested positive for at least one pathogen. Pediatric patients represented 72.5% of positive cases. Rhinovirus/enterovirus (32%) was the most prevalent, followed by SARS-CoV-2 (16%), respiratory syncytial virus (RSV; 13%), and adenovirus (10%).

**Conclusions:**

The study underscores the significant seasonality and age-specific prevalence of RTIs, with winter peaks and a high incidence of rhinovirus/enterovirus, SARS-CoV-2, RSV, and adenovirus. These results emphasize the necessity of ongoing surveillance and targeted public health interventions to manage RTIs effectively.

## 1 Background

Respiratory tract infections (RTIs) are one of the most common infections and are a leading cause of morbidity and mortality worldwide. They can cause disease in all age groups; nonetheless, children < 5 years old, older adults, and individuals with underlying morbidities are among the most vulnerable. Acute respiratory failure and acute respiratory distress syndrome are serious conditions that can be brought on by respiratory infections. While respiratory viruses such as rhinovirus, parainfluenza viruses, adenovirus, human bocavirus, and seasonal coronaviruses are known to cause mild upper respiratory tract infections, influenza A and B viruses and respiratory syncytial virus (RSV) account for the majority of deaths and hospitalizations.

The COVID-19 pandemic has profoundly impacted the epidemiology of respiratory viruses due to widespread public health interventions such as lockdowns, mask mandates, and social distancing (1–5). Several international studies have examined RTI patterns during the COVID-19 era. Reports from Taiwan and Switzerland found that, in addition to SARS-CoV-2, rhinovirus/enterovirus remained the dominant pathogen despite extensive public health measures, whereas influenza detection declined significantly (1, 3). In contrast, a study conducted in Turkey observed a sharp drop in influenza cases during the pandemic, indicating regional variability in RTI trends (6). A systematic review of global RTI prevalence also highlighted the sustained circulation of rhinoviruses and enteroviruses, which were less affected by pandemic interventions than other respiratory viruses (7). In the Middle East, RTI epidemiological data remain scarce. Studies from Lebanon and Qatar have reported high rates of rhinovirus, influenza, and adenovirus infections among hospitalized patients (8, 9).

Previous research from Saudi Arabia has focused primarily on pediatric RTI cases, with findings indicating that RSV and adenovirus were the most commonly detected pathogens in children (10–12). However, few studies have comprehensively analyzed RTI epidemiology across all age groups in the region, particularly during the COVID-19 era.

The seasonality of respiratory infections is a well-documented phenomenon and is impacted by a number of variables, such as human behavior, virus features, and environmental influences (10). In temperate regions, infections like influenza and the common cold peak in winter, while in tropical areas, patterns vary, often aligning with rainy seasons (11, 12). Cold temperatures and low humidity enhance viral stability and transmission, while increased indoor activity facilitates spread. Additionally, viruses like influenza and RSV undergo genetic drift and shift, driving seasonal outbreaks as immunity wanes (13).

In Saudi Arabia, respiratory infection seasonality is shaped by its unique climate and cultural events, such as the annual Hajj pilgrimage. A study of children with respiratory tract infections in Riyadh (2013–2014) found viral pathogens in 24% of cases, with RSV being most common and peaking in winter.

This study demonstrated the importance of viral pathogens in RTIs, noting a peak in virus detection during the winter months (14).

This emphasizes the need for continuous surveillance and tailored public health interventions, especially considering the country's role as a host for large-scale religious gatherings, which poses unique challenges for disease control and prevention.

In this study, the etiology and epidemiological parameters of RTIs, including age and seasonality, in all patients tested using multiplex polymerase chain reaction (PCR) in King Abdulaziz Medical City in Riyadh, Saudi Arabia, between January 2021 and December 2022 were investigated. We aimed to evaluate the age, gender, pathogen distribution, and seasonality of respiratory pathogens during the COVID-19 pandemic.

## 2 Materials and methods

King Abdulaziz Medical City (KAMC) is part of the Ministry of National Guard Health Affairs and is an advanced 2,500-bed tertiary health facility located in Riyadh, Saudi Arabia. Respiratory specimens, including nasopharyngeal swabs, bronchoalveolar lavage, sputum, and tracheal aspirates, were collected from patients presenting to our hospital with respiratory illness from January 2021 to December 2022 and tested using the BioFire Filmarray Respiratory Panel 2.1 plus (RP2.1 plus) kit (BioFire^®^Diagnostics, Salt Lake City, UT, USA) run on the BioFire Torch System instrument as per the manufacturer's instructions. The RP2.1 plus kit is intended for the detection and differentiation of nucleic acid from the following organisms: Adenovirus, coronavirus 229E, coronavirus HKU1, coronavirus NL63, coronavirus OC43, Middle East respiratory syndrome coronavirus (MERS-CoV), severe acute respiratory syndrome coronavirus 2 (SARS-CoV-2), human metapneumovirus, human rhinovirus/enterovirus, influenza A virus, influenza A virus A/H1, influenza A virus A/H3, influenza A virus A/H1-2009, influenza B virus, parainfluenza virus 1, parainfluenza virus 2, parainfluenza virus 3, parainfluenza virus 4, respiratory syncytial virus, *Bordetella parapertussis, Bordetella pertussis, Chlamydia pneumoniae*, and *Mycoplasma pneumoniae*. The BioFire RP2.1 Plus assay utilizes a nested set multiplex PCR to amplify and detect nucleic acid sequences from multiple respiratory pathogens in a single test. This method integrates sample preparation, nested amplification, and detection into a streamlined process, enabling rapid and accurate identification of viral and bacterial targets directly from patient specimens. The BioFire RP2.1 Plus assay has demonstrated high sensitivity and specificity across multiple studies (15–18). According to a study by Leber et al., the assay shows a sensitivity range of 95–100% and specificity exceeding 99% for most of the pathogens it tests (17). In terms of cross-reactivity, the design of the BioFire RP2.1 Plus assay minimizes this risk by using unique primer and probe sets that are highly specific to target pathogens. Independent evaluations, such as those conducted by Poritz et al., have shown minimal cross-reactivity, confirming the assay's robustness against potential diagnostic errors (15).

In this retrospective study, we reviewed our laboratory records from KAMC for samples that were tested during the period from January 2021 to December 2022. The study employed a convenience sampling approach, as respiratory specimens were included based on their availability in the hospital's records and their alignment with the study's inclusion criteria. The etiology and epidemiological parameters that were examined, were age, gender, pathogen detected. and seasonality.

A total of 19,509 (7,484 in 2021 and 12,019 in 2022) respiratory specimens were included in this study. To address potential biases from repeat testing, samples collected from the same patient within a 30-day period were excluded to ensure each data point represented a unique infection event. The study was approved by the King Abdullah International Medical Research Center's (KAIMRC) institutional review board (IRB) (Approval No: IRB/0945/23). The study employed descriptive statistical methods to elucidate the frequency and distribution of respiratory pathogens identified in the specimens. Data were tabulated and visualized using Microsoft Excel (Microsoft Corporation. Redmond, Washington, United States) and PowerBI (Microsoft Corporation. Redmond, Washington, United States)., which facilitated the generation of pie and rainbow charts to illustrate the distribution of pathogens across different times and demographic groups.

## 3 Results

**Figure 1:**
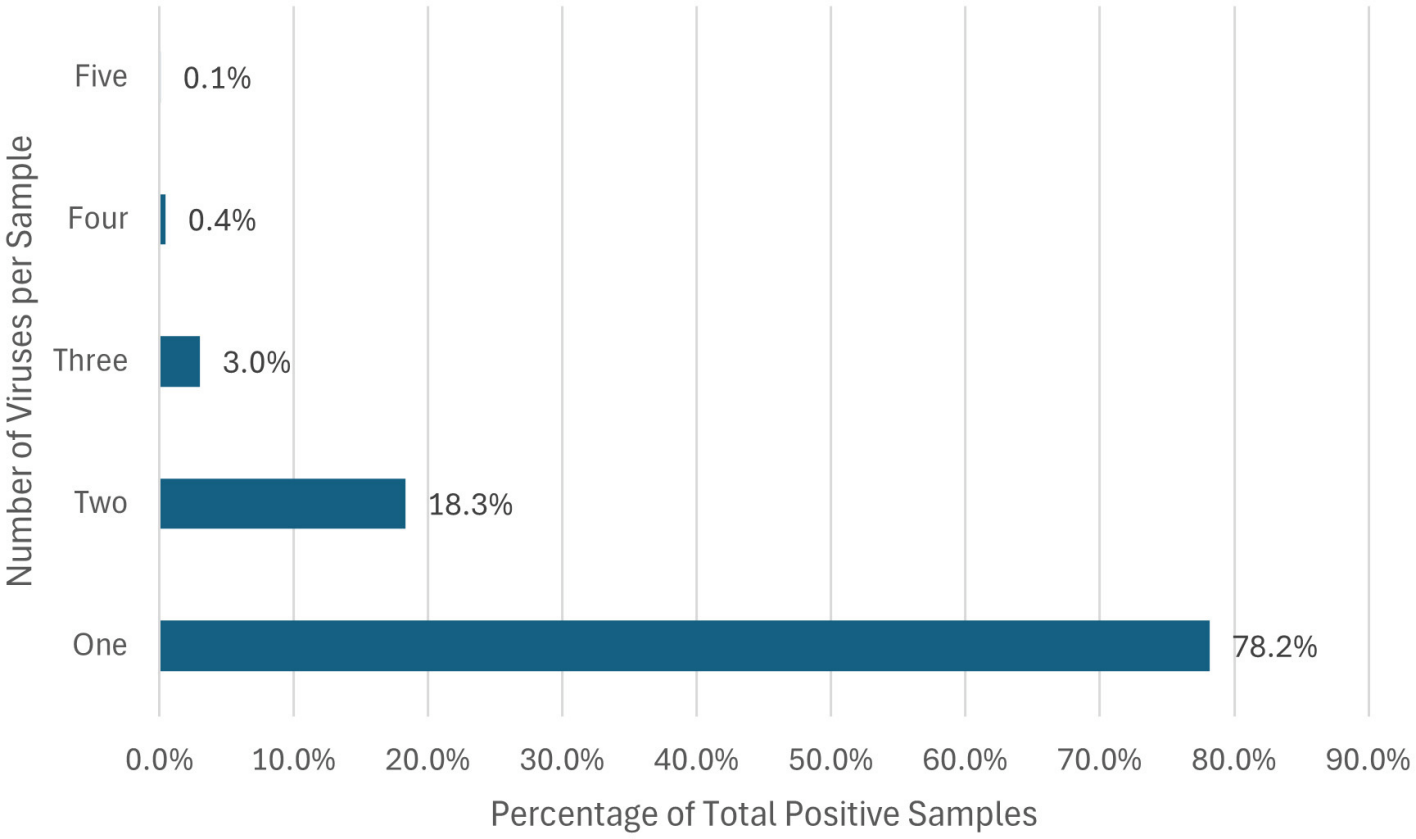
Percentage of respiratory co-infections. This horizontal bar chart represents the distribution of the percentage of different organisms identified per positive sample.

A total of 19,509 respiratory samples across 2021 and 2022 were tested. The demographic distribution of the patients, detailed in Table 1, revealed a predominance of pediatric patients, comprising 72.5% of the study population. Regarding gender distribution, males represented 53.9% (5,612) and females 46.1% (4,794) of the cases. Of the specimens, 53.3% (10,406) tested positive for at least one pathogen. Most of the samples were positive for one pathogen (78.2%), while co-infections were seen in the remainder of the samples (21.8%), where 18.3% had two pathogens, 3% had three pathogens, and < 1% had four or more pathogens (Figure 1). The data on co-infections revealed layered complexity within respiratory infections. While most patients had mono-infections, the presence of multiple pathogens in over a fifth of the positive cases presents a challenge for treatment due to potential interactions between pathogens and their cumulative effect on the patient's condition.

**Table 1:** Demographic characteristics and frequency of viral respiratory infections by age group.

**Demographic**	**Number/frequency**
Males	5,612 (53.9%)
Females	4,794 (46.1%)
**Age group (in years)**	
0 to < 6 months	1,693 (16.2%)
6 months to 2 years	3,137 (30.1%)
3 to 7 years	1,944(18.6%)
8 to 17 years	795 (7.6%)
18 to 64 years	1,380 (13.2%)
≥ 65 years	1,457 (14%)

### 3.1 Etiology of respiratory infections

**Figure 2:**
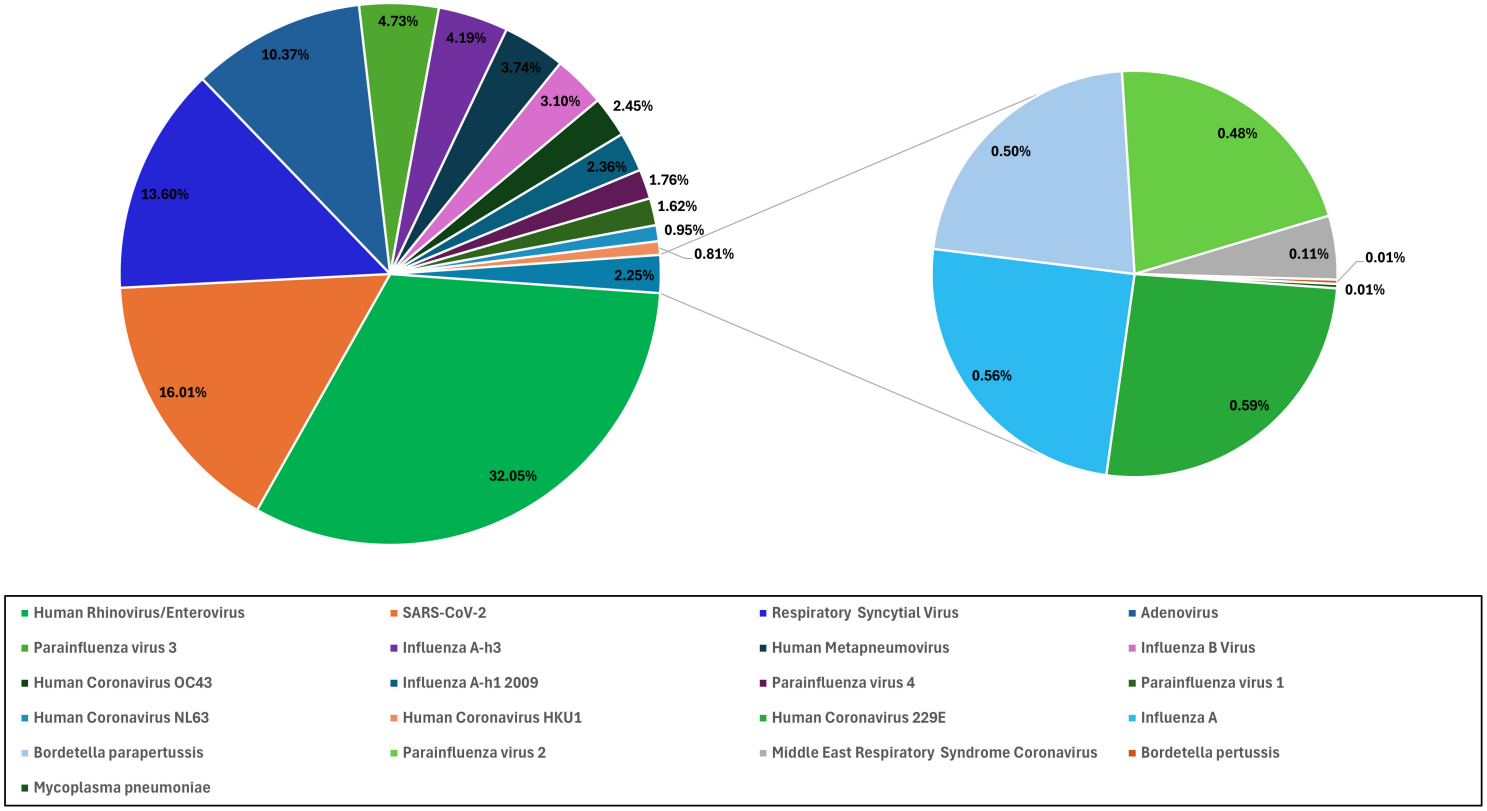
The percentage of different respiratory viruses. The proportional distribution of positive samples for various respiratory pathogens.

Over the 2-year period in which this study was conducted, rhinovirus/enterovirus were the predominant pathogens throughout, comprising 32% (4,199) of all positive samples, followed by SARS-CoV-2 (2097, 16%), respiratory syncytial virus (RSV) (1,782, 13%), and adenovirus (1,358, 10%; Figure 2). There were 931 (7%) cases of influenza A virus, 549 of which were caused by the influenza A h3 variant, while 309 cases were caused by the influenza A h1-2009 variant, and 73 cases were attributed to non-typeable influenza A (Figure 2). There were 1,126 (8.5%) parainfluenza virus cases. Parainfluenza 3 was the most common parainfluenza (620, 55%) of all parainfluenza cases, followed by parainfluenza 4 and 1 [231 (20.5%) and 212 (18.8%), respectively], and there were only 63 cases (5.5%) of parainfluenza 2 (Figure 2).

### 3.2 Distribution of respiratory infections by age group

**Figure 3:**
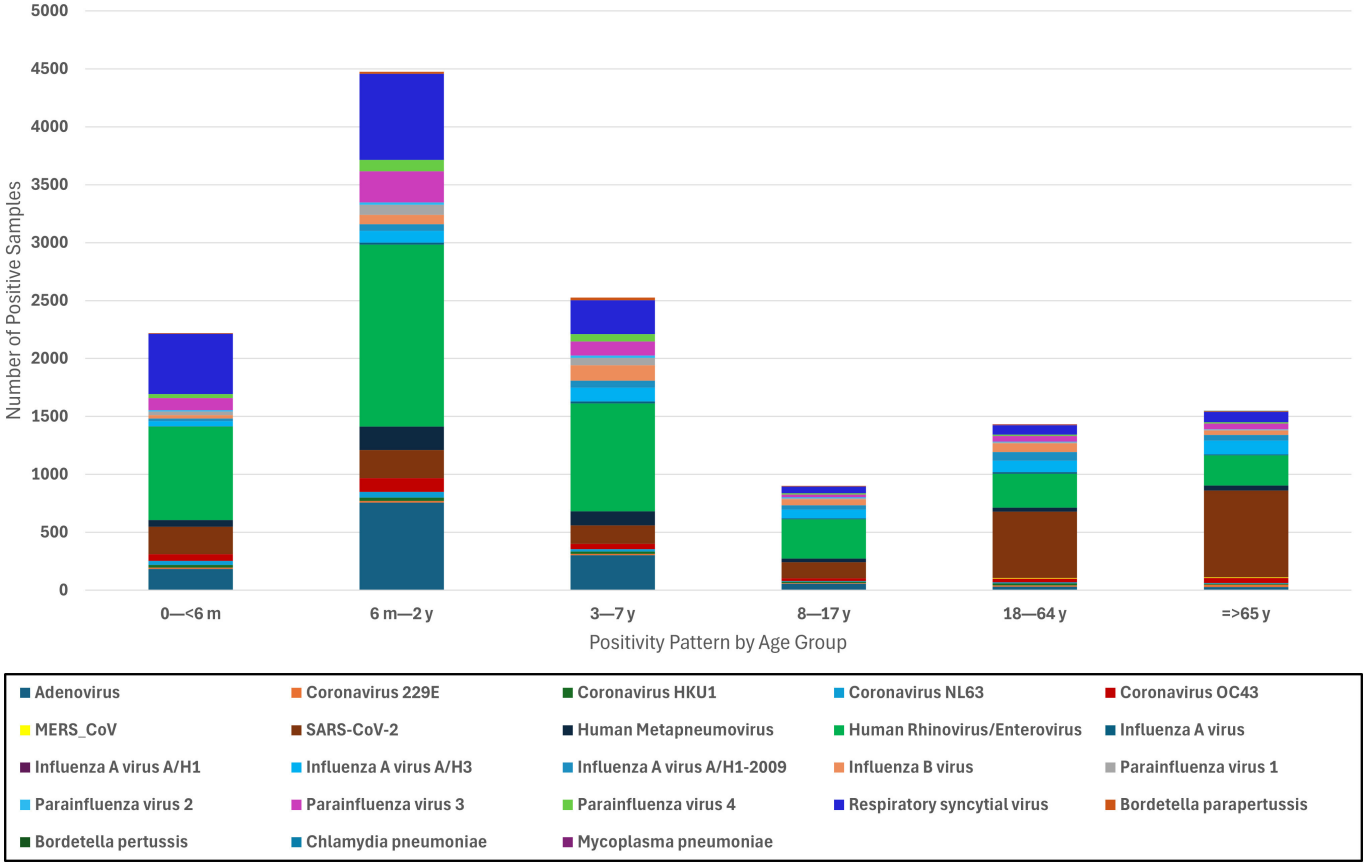
Distribution of respiratory pathogens by age group. This stacked bar chart presents the distribution of positive samples for various respiratory pathogens across different age groups.

The highest positivity rates were observed in pediatric patients (72.5%), with the age group from 6 months to 2 years old presenting the highest positivity rate (30.1%; Figure 3). This suggests a higher vulnerability or exposure within this demographic. Conversely, the 8–17-year-old age group showed the lowest positivity rates among all age groups (7.6%; Figure 3). This could be partially explained by the completion of childhood vaccinations.

When clustered by age groups, rhinovirus/enterovirus remained the most prevalent pathogen among the pediatric groups (< 6 months, 6 months to 2 years, 3–7 years, and 8–17 years). In the < 6 months demographic, RSV was the second most prevalent pathogen (522, 23.5%), followed by SARS-CoV-2 (238, 10%; Figure 3). Despite a large difference in their positivity rate, those aged 6 months to 2 years and 3–7 years followed a similar pattern, where adenovirus infections were the second most common etiological agent of respiratory disease, trailed closely by RSV. The majority of infections in the adult (18–64 years) and older adult (≥65 years) populations were caused by SARS-CoV-2 (572, 39%) and then rhinovirus/enterovirus and influenza A infections. Notably, all 15 MERS-CoV cases occurred in adult patients (Figure 3).

### 3.3 Seasonal distribution

**Figure 4:**
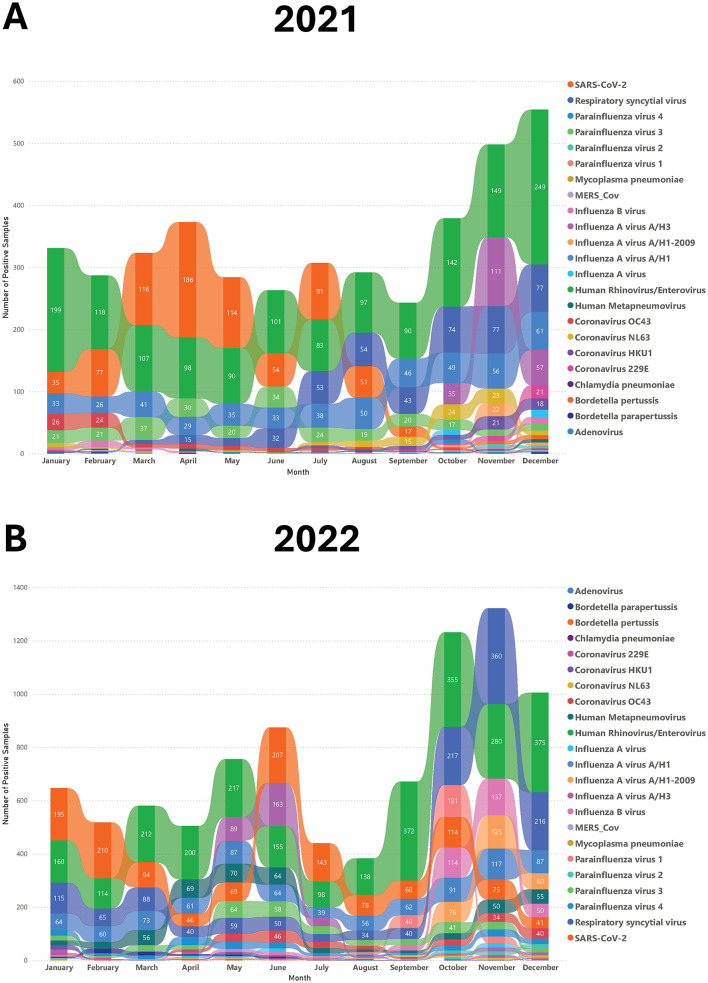
The seasonal pattern of different respiratory viruses in 2021 and 2022. The rainbow chart shows monthly positive samples for various respiratory pathogens in 2021 **(A)** and 2022 **(B)**.

The monthly number of positive cases for each respiratory pathogen is illustrated in Figure 4. Starting in October, there was a sharp increase in the number of cases, with rhinovirus/enterovirus, RSV, and SARS-CoV-2 being the dominant etiologies during these months (Figure 4). During the month of November, SARS-CoV-2 was overtaken by influenza and adenovirus infections, with influenza B being the predominant influenza subtype, followed by influenza A H1-2009, and then adenovirus. Rhinovirus/enterovirus and SARS-CoV-2 consistently exhibited a constant presence, showing the highest number of cases from January to September, with SARS-CoV-2 peaking at its highest levels in January and February and peaking again in June and July (Figure 4). The differences in seasonal distribution and number of infections between 2021 and 2022 could be partially explained by COVID-19 restrictions in Saudi Arabia. COVID-19 restrictions, including mandatory mask-wearing, were officially lifted on June 13, 2022. This explains the increase in respiratory infections following this restriction lift (Figure 4).

## 4 Discussion

This study provides a comprehensive analysis of the epidemiology and seasonal patterns of respiratory infections in Saudi Arabia during the COVID-19 era, utilizing multiplex PCR technology. By examining data from 19,509 respiratory samples collected between 2021 and 2022, we identified key trends in pathogen prevalence, age-specific susceptibility, and seasonal dynamics. The findings underscore the significant burden of respiratory infections, particularly among pediatric populations, and underscore the impact of public health interventions, such as COVID-19 restrictions, on the transmission of respiratory viruses.

Our study revealed a pediatric predominance, with 72.5% of samples coming from children, and a slight male majority (53.9%). Overall, 53.3% of samples tested positive for at least one pathogen, with single infections accounting for 78.2% of cases and co-infections for 21.8%. These findings align with global trends observed during the COVID-19 pandemic, particularly the increased prevalence of co-infections following the easing of pandemic restrictions. For instance, Maison et al. reported a rise in co-infections among pediatric patients post-lockdown, with single infections remaining predominant but multiple viral detections becoming more common in 2021 and 2022 compared to pre-pandemic levels (19). This similarity in findings underscores the broader epidemiological shifts during the pandemic where increased interactions and subsequent viral exposures may have facilitated higher rates of co-infections.

The most frequently detected pathogens in our study were rhinovirus/enterovirus (32%), SARS-CoV-2 (16%), RSV (13.6%), and adenovirus (10.3%). Rhinovirus/enterovirus was particularly prevalent in pediatric patients, especially those aged 6 months to 2 years, while SARS-CoV-2 was more common in adults and the adults aged 65 years and older. These findings are consistent with global patterns reported during the pandemic, where rhinoviruses/enteroviruses remained highly prevalent despite public health measures aimed at curbing respiratory virus transmission. Schüz et al. noted similar trends in their meta-analysis, emphasizing the resilience of these viruses in the face of widespread interventions (7). Furthermore, the systematic review and meta-analysis by Dallmeyer et al. revealed that rhinovirus/enterovirus and RSV were the most frequent pathogens detected in pediatric patients, particularly among younger age groups, underscoring the significant burden of these infections even during a period dominated by SARS-CoV-2 (20). The presence of these viruses in younger pediatric cohorts illustrates the significant burden of these infections, even during a period characterized by widespread public health interventions aimed at reducing the spread of SARS-CoV-2.

Our findings are consistent with global observations, though regional variations exist. For instance, a study from Qatar, reported influenza as the leading cause of influenza-like illnesses (ILIs) among adults, followed by rhinovirus and seasonal coronaviruses, with RSV and human metapneumovirus (HMPV) showing strong winter peaks (8). Similarly, Chen et al. found that rhinovirus/enterovirus was the most common pathogen in Taiwan during the pandemic, followed by adenovirus and SARS-CoV-2, with co-infections accounting for 5.3% of cases (1). A report from a tertiary hospital in Singapore during the early phase of the COVID-19 outbreak identified rhinovirus as the leading cause of community-acquired respiratory infections requiring hospitalization, followed by influenza and seasonal coronaviruses (5). These results underscore differences in etiological distribution and detection methods across regions, while also aligning with our observations and highlighting the global predominance of rhinoviruses, even during a pandemic dominated by SARS-CoV-2.

The COVID-19 pandemic significantly altered the epidemiology of respiratory infections. In Saudi Arabia, comprehensive restrictions were implemented early in the pandemic and gradually lifted by June 2022. Our study period (2021–2022) captured both the height of these measures and their subsequent relaxation. Notably, we observed a marked increase in respiratory tract infections (RTIs) following the easing of restrictions, particularly in the latter half of 2022. This rise in RTIs, driven by pathogens such as rhinovirus/enterovirus, RSV, and adenovirus, underscores the impact of public health policies on disease transmission dynamics. Reduced social distancing and preventive measures likely facilitated increased viral spread, a trend mirrored globally as countries relaxed COVID-19 restrictions. Pre-pandemic studies in Saudi Arabia pointed out the significant burden of respiratory infections, particularly among children. A 2017 study reported over five million cases of acute respiratory infections from 2012 to 2013, with more than 60% occurring in infants under 1 year old. Similarly, our study found that 46% of infections occurred in children under 2 years old, with rhinovirus/enterovirus and RSV being the most common pathogens. These findings suggest a persistent epidemiological profile for these viruses over the years (21). More recent studies from Saudi Arabia and neighboring regions have corroborated these trends. For example, a study from Riyadh involving 503 pediatric patients under 5 years old identified RSV and influenza as the most prevalent viruses, consistent with our findings. However, the limited scope of PCR assays used in some studies, which excluded pathogens like rhinovirus, may have underestimated their prevalence (22). Farrag et al. also emphasized the impact of mass gatherings, such as the Hajj pilgrimage, on respiratory virus transmission, with rhinoviruses and influenza viruses being the most common pathogens detected in Hajj returnees (23). These findings align with global trends, where similar rises in RTIs were documented post-COVID-19 restrictions, shedding light on the dual impact of pandemic policies on both COVID-19 and other respiratory infections.

Seasonal patterns were also evident, with respiratory infections peaking in the colder months. Influenza and adenovirus cases surged in November, while RSV activity increased from October, peaking in winter. These observations align with studies from other regions, such as Rousogianni et al., who reported similar winter peaks for influenza A and adenovirus in Greece (24). The U.S. CDC reported that RSV epidemics typically peak during winter, a pattern initially disrupted by the COVID-19 pandemic but later returning to its usual seasonal regularity (25). Similarly, a report from the United Kingdom noted an increase in RSV cases during winter, further corroborating our findings (26). A review by Moriyama et al. highlighted that influenza, human coronaviruses, and RSV are typically winter viruses, while rhinoviruses, adenoviruses, and human metapneumoviruses circulate year-round (10). These findings correlate with our study, where rhinovirus/enterovirus was detected throughout the year, and influenza and RSV peaked in winter. However, our observation that RSV activity began earlier (October) and declined by February differs from a 2013–2014 Saudi Arabian study, which reported RSV peaks from December to March. This discrepancy may stem from differences in detection methods (multiplex PCR vs. DFA) and factors such as enhanced surveillance, increased public awareness, and improved healthcare access, all of which can influence epidemic timing and intensity (14). Nonetheless, these consistent observations across different regions underscore the robust seasonal dynamics of respiratory viruses, particularly RSV, and validate the winter peaks identified in our study. Saudi Arabia's arid climate, characterized by extreme temperatures and low humidity, may contribute to the observed winter peaks. Lower humidity in colder months could enhance viral stability and airborne transmission, while increased indoor crowding during winter likely facilitates spread. Future studies might further our understanding by incorporating meteorological data with infection rates to give a fuller analysis of the factors affecting respiratory virus transmission.

The significance of this research lies in its examination of the distribution and seasonality of respiratory pathogens at a time when global health systems were predominantly oriented toward managing COVID-19. Particularly novel is the study's illumination of the seasonal distribution of these pathogens, and to our knowledge the first and largest study from the middle east to report the frequency and distribution of respiratory pathogens using the Filmarray multiplex PCR technology, providing critical data that diverge from the patterns noted in previous research conducted prior to the pandemic (8, 9, 21, 23). This study has several limitations. As a retrospective analysis using a single multiplex PCR assay, it does not capture co-infections with pathogens outside the Filmarray respiratory panel. Additionally, the study was conducted at a single center, although the large sample size (over 19,000 samples) strengthens its validity. The lack of clinical severity markers, such as ICU admissions or mortality rates, limits our ability to assess the disease burden associated with detected pathogens. Future prospective studies integrating laboratory data with clinical outcomes are essential to fully understand the public health impact of respiratory infections.

Our findings have significant implications for public health strategies in Saudi Arabia. The observed winter peaks of RSV, influenza, and adenovirus suggest that vaccination campaigns should be prioritized in early autumn, particularly for high-risk groups such as infants, older adults, and immunocompromised individuals. Enhanced surveillance during mass gatherings, such as the Hajj pilgrimage, could further mitigate transmission risks. Additionally, understanding seasonal trends can help health systems optimize resource allocation, staff levels, and medical supplies during peak transmission periods.

The prominence of rhinovirus/enterovirus and RSV in pediatric populations underscores the need for targeted interventions, such as earlier and more frequent vaccinations for young children. Public health communications should also emphasize the importance of preventive measures, especially during peak seasons, to reduce the burden of respiratory infections.

This study provides critical insights into the epidemiology of respiratory viruses in Saudi Arabia during the COVID-19 era, delineating the prevalence, seasonality, and demographic distribution of key pathogens. By leveraging multiplex PCR technology, we have identified patterns that diverge from pre-pandemic studies, reflecting the unique impact of COVID-19 on respiratory virus transmission. These findings fill important knowledge gaps and offer a foundation for refining public health strategies, improving disease surveillance, and enhancing clinical management of respiratory infections in Saudi Arabia. Future research integrating environmental and clinical data will further advance our understanding of respiratory virus dynamics and inform more effective interventions.

## Data Availability

The data supporting the findings of this study are not publicly available due to restrictions imposed by the King Abdullah International Medical Research Center (KAIMRC). According to KAIMRC policies, patient data and related clinical information cannot be shared publicly to protect patient privacy and confidentiality. However, specific data details may be available from the corresponding author upon reasonable request and with permission from KAIMRC, subject to institutional and ethical guidelines. Requests to access the datasets should be directed to Alzahranin@ksau-hs.edu.sa.
